# *Allium subhirsutum* L. as a Potential Source of Antioxidant and Anticancer Bioactive Molecules: HR-LCMS Phytochemical Profiling, In Vitro and In Vivo Pharmacological Study

**DOI:** 10.3390/antiox9101003

**Published:** 2020-10-16

**Authors:** Riadh Badraoui, Tarek Rebai, Salem Elkahoui, Mousa Alreshidi, Vajid N. Veettil, Emira Noumi, Khaled A. Al-Motair, Kaïss Aouadi, Adel Kadri, Vincenzo De Feo, Mejdi Snoussi

**Affiliations:** 1 Department of Biology, University of Hail, P.O. Box 2440, Ha’il 81451, Saudi Arabia; salemelkahoui@gmail.com (S.E.); mousa.algladi@gmail.com (M.A.); vajidnv@gmail.com (V.N.V.); eb.noumi@uoh.edu.sa (E.N.); 2 Section of Histology-Cytology, Medicine Faculty of Tunis, University of Tunis El Manar, 1007 La Rabta, Road Djebal Lakhdhar, Tunis 1007, Tunisia; 3 Department of Histo-Embryology and Cytogenetics, Medicine Faculty of Sfax, University of Sfax, Road of Majida Boulia, Sfax 3029, Tunisia; tarek.rebai@fmsf.rnu.tn; 4 Laboratory of Bioactive Substances, Center of Biotechnology of Borj Cedria (CBBC), BP 901, Hammam Lif 2050, Tunisia; 5 Laboratory of Bioressources: Integrative Biology & Recovery, High Institute of Biotechnology-University of Monastir, Monastir 5000, Tunisia; 6 Molecular Diagnostic and Personalized Therapeutics Unit, University of Ha’il, Ha’il 81451, Saudi Arabia; kh.almutayr@uoh.edu.sa; 7 Department of Chemistry, College of Science, Qassim University, Buraidah 51452, Saudi Arabia; kaiss_aouadi@hotmail.com; 8 Laboratory of Heterocyclic Chemistry, Natural Products and Reactivity, Department of Chemistry, Faculty of Science of Monastir, University of Monastir, Monastir 5019, Tunisia; 9 Department of Chemistry, College of Science and Arts in Baljurashi, Albaha University, Albaha 65527, Saudi Arabia; lukadel@yahoo.fr; 10 Department of Chemistry, Faculty of Science of Sfax, University of Sfax, BP 1117, Sfax 3000, Tunisia; 11 Department of Pharmacy, University of Salerno, Via Giovanni Paolo II, 132, Fisciano, 84084 Salerno, Italy; defeo@unisa.it; 12 Laboratory of Genetics, Biodiversity and Valorisation of Bioresources, High Institute of Biotechnology-University of Monastir, Monastir 5000, Tunisia

**Keywords:** anticancer, angiogenesis, antioxidant, apoptosis, bone metastases, *Allium subhirsutum*, phytochemistry, cell proliferation

## Abstract

This study investigated *Allium subhirsutum* L. (AS) anticancer and antioxidant effects and inhibition of tumor angiogenesis in a murine model of skeletal metastases due to inoculation of Walker 256/B cells. Phytochemical composition of AS extract (ASE) was studied by High Resolution-Liquid Chromatography Mass Spectroscopy (HR-LCMS). Total phenolic and flavonoid contents (TPC and TFC) were determined. In vitro, the antioxidant properties were evaluated by reducing power and antiradical activity against DPPH. Cancer cells’ proliferation, apoptosis, metastatic development and angiogenesis were evaluated using Walker 256/B and MatLyLu cells. The p-coumaric acid was the major phenolic acid (1700 µg/g extract). ASE showed high levels of TPC and TFC and proved potent antioxidant effects. ASE inhibited Walker 256/B and MatLyLu cells’ proliferation (Half-maximal inhibitory concentration: IC_50_ ≃ 150 µg/mL) and induced apoptosis. In silico and in vivo assays confirmed these findings. ASE effectively acts as a chemo-preventive compound, induces apoptosis and attenuates angiogenesis and osteolytic metastases due to Walker 256/B malignant cells.

## 1. Introduction

Cancer is categorized as one of the most important causes of death all over the world [1]. It is associated with serious clinical implications together with social and economic impacts [2]. The non-controlled hyper-proliferation associated with cancer diseases includes several hallmarks, such as the escape from cell death by apoptosis, invasion, metastatic potential and angiogenesis [3].

Malignant Walker 256/B and MatLyLu cells have been used in animal models to study interactions between cancer and bone, they are very reproducible methods to induce osteolytic and osteosclerotic metastases, respectively [4,5,6]. Furthermore, an enhanced angiogenesis and a downregulation of apoptosis have been outlined during malignant processes [7,8]. Earlier reports, including our studies, indicate that these cancerous cells, with high bone trophicity, induce bone loss, disrupt osteoclasts (the bone-resorbing cells) cyto-morphometry and enhance angiogenesis in vicinity of the bone metastatic foci [9,10,11]. The two used cell lines are poorly immunogenic and commonly used for murine models of bone osteolytic and osteosclerotic metastasis, respectively [5,12,13].

It is well known that plant products and some vegetables, used as dietary supplements, might significantly reduce the effects of cancer proliferation. Hence, ethno-medicinal plants had and still have a tremendous contribution in the development of several drugs to treat and/or prevent several diseases, including the different stages of cancer [14]. Their protective and/or alleviating effects might induce a decrease in cell proliferation and reduce cancer invasion and spread. Nevertheless, it has been proposed that the whole-plant effects might be much better than its active components [15].

Garlic species have been and still are used as functional food and folk remedy due to their health beneficial properties [16]. Investigations conducted on garlic species, for pharmacological effects, showed that garlic active principles are mainly cysteine, sulphoxides, flavone, tannins, etc. [17]. Furthermore, biochemical analysis of different garlic extracts by Liquid Chromatography-Mass Spectrometry (LC-MS/MS) showed their rich profile in bioactive compounds.

Bulbs of *Allium subhirsutum* (AS) were investigated in terms of phenolic profile and it has been shown that it is rich with phenolic acids [16]. The authors concluded that not only did it possess different bioactive compounds but also, they encourage its consumption. So far, AS might be functional food sources of biologically effective phenolic and high levels of phenols. Garlic plants, including hairy garlic, have been used for medical purposes through the recorded history [18]. The medical use and efficacy of garlic in treating diseases, including cancer, were reported throughout the ages [18]. Craig, in 1999 [19], reported the traditional use of Allium plants, including *A. subhirsutum,* in both nutrition and treatment of diseases. The known benefits include anticancer and chemo-preventive effects in several cancers with bone metastatic potential, including prostate cancer [20,21]. Epidemiological studies reported reduced risk of several types of cancers with the intake of allium products [22]. The distinctive compounds of allium species, particularly organosulfur, explained its traditional use and their interactions with cancer diseases [20,23].

Previous studies showed that Alliaceae have promising contents and are of great importance owing to their versatile uses as antioxidants, flavoring agents, fragrance and therapeutics [17]. However, the biological activities of AS extract (ASE), particularly its antioxidant and anticancer activities, are not well documented and need to be further explored. To the best of our knowledge, no study reported the effect of AS on angiogenesis and malignant cells-induced bone metastases. As little is known about the effects of AS on high osteophilic and metastasizing cells, the aim of this work was to extend additional knowledge and understanding of AS anticancer effects in two cancerous cell lines: Walker 256/B and MatLyLu. The study involves assessment of total phenolic and flavonoid contents, antioxidant activity and both in vitro and in vivo anticancer assays.

## 2. Materials and Methods

### 2.1. Chemicals

Dulbecco’s modified Eagle’s medium (DMEM), penicillin, streptomycin sulphate and sodium pyruvate were obtained from Eurobio (Les Ulis, France). Fetal calf serum, isoflurane and nonessential amino acids were purchased from Seromed Biochrom (Berlin, Germany), AErrane (Baxter S.A., Belgium) and Cambrex (Walkersville, MD, USA), respectively. Other chemicals, such as trichloroacetic acid (TCA), quercetin, ferric chloride, potassium ferricyanide (K_3_Fe(CN)_6_), gallic acid, etc., were of analytical grade and purchased from Sigma-Aldrich.

### 2.2. Plant Material and Preparation

AS was collected, in May 2019, from Makkah. It was identified by Dr. Mseddi K. Its Voucher specimen number is 7427 (H. Yildirim, deposited in EGE University’s Herbarium). The extract used in the current study was prepared from the bulbs using the 3-times methanol extraction method. The extract was pooled and filtered, then stored (in Eppendorf tube at 25 °C maximum) until further use. The total phenolic and flavonoid contents of the extract were evaluated using spectrophotometric methods as previously described with slight modifications [24,25]. The total phenolic content (TPC) was expressed as gallic acid equivalent (mg GAE/g extract). However, the total flavonoid content (TFC) was expressed as quercetin equivalent (mg QE/g extract).

### 2.3. Identification of Bioactive Compounds by High Resolution-Liquid Chromatography Mass Spectroscopy (HR-LCMS)

Phytochemical analysis ASE was assessed using Ultra High-performance liquid chromatography Photodiode-Array Detector 323 Mass Spectrophotometer (HR-LCMS 1290 Infinity UHPLC System), Agilent 324 Technologies^®^, USA. The liquid chromatographic system consisted of a HiP sampler, binary gradient solvent pump, column compartment and Quadrupole Time of Flight Mass Spectrometer (MS Q-TOF) with a dual Agilent Jet Stream Electrospray (AJS ES) ion source. 10 μL of sample was injected into the system, followed by separation in SB-C18 column (2.1 × 50 mm, 1.8 μm particle size). 1% formic acid in deionized water (solvent A) and acetonitrile (solvent B) were used as solvents. Flow rate of 0.350 mL/min was used, while MS detection was performed in MS Q-TOF. Compounds were identified via their mass spectra and their unique mass fragmentation patterns. Compound Discoverer 2.1, ChemSpider and PubChem were used as the main tools for the identification of the phytochemical constituents.

### 2.4. In Silico ADME and Toxicity Profiles

The physicochemical and pharmacokinetics properties of the selected peptides were estimated using ADME (absorption, distribution, metabolism and excretion) descriptors by a SwissADME online server (http://www.swissadme.ch/). An online ProTox-II webserver (http://tox.charite.de/tox/) was also used to explore the toxicity profiles (hepatotoxicity, immunotoxicity, genetic toxicity endpoints, especially cytotoxicity, mutagenicity and carcinogenicity) of the selected peptides as previously reported [26].

### 2.5. Antioxidant Assays

#### 2.5.1. Reducing Power Assay

The reducing power was monitored according to a previously published method [27]. Increased concentrations of the extract (100, 200, 500, 750, 1000 µg/mL) were mixed with 2.5 mL of 200 mmol/L sodium phosphate buffer (pH 6.6) and 2.5 mL of 1% potassium ferricyanide. The mixture was shaken, then incubated for 20 min at 50 °C. After incubation, 2.5 mL of trichloroacetic acid (10%, *w*/*v*, in water) was added and then the mixture was centrifuged at 1000 rpm for 8 min. 2.5 mL of the supernatant was then mixed with 2.5 mL of distilled water and 0.5 mL of 1% ferric chloride. The absorbance was measured spectrophotometrically at 700 nm. The extract concentration providing 0.5 of absorbance was calculated from the graph of absorbance registered at 700 nm against the correspondent extract concentration.

#### 2.5.2. Antiradical Activity against DPPH

The capacity to scavenge the free-radical 2, 2-diphenyl-1-picrylhydrazyl (DPPH) was assessed according to a previously published method [14,28] and using ascorbic acid as standard solution. Different concentrations of the AS extract (1, 10, 100, 200 g/mL) have been added to the DPPH solution. Loss of DPPH pigment was assessed spectrophotometrically at 515 nm. IC_50_ was calculated from the graph after registering Optical Density (OD) values.

### 2.6. In Vitro Assays

#### 2.6.1. Malignant MatLyLu and Walker 256/B Cell Lines and Culture Conditions

Malignant MatLyLu and Walker 256/B cells were used to assess the in vitro anticancer activity of ASE. Both MatLyLu and Walker 256/B malignant cells are known to metastasize to bone and induce differential effects on its remodeling and/or metabolism. Bone turnover is a hallmark of these MatLyLu prostate cancer and Walker 256/B mammary gland cancer cells.

Cells were grown in suspension in DMEM supplemented with 5% fetal calf serum, 100 IU/mL of penicillin, 100 g/mL of streptomycin sulphate, 1% of non-essential amino acids and 1 mM of sodium pyruvate. Cells were seeded on 96-well culture plates and incubated for 72 h at 37 °C in a humidified atmosphere using a water-jacketed 5% CO_2_, as previously published [4,10].

#### 2.6.2. In Vitro Anticancer Assessment and MTT Assay

The dye compound 3-(4,5-Dimethylthiazol-2-yl)-2,5-diphenyltetrazolium bromide (MTT) assay was performed by quantitative colorimetric method as suggested by Sylvester [29] with little modifications. Malignant Walker 256/B and MatLyLu (5 × 10^2^ cells/well) were seeded on 96-well plates with or without ASE. Anti-cancer activity of medicinal plants was determined on cell lines at various ASE concentrations (50, 100, 150, 200 μg/mL). Pure (100%) H_2_O_2_ was used as a positive control. At the appropriate time (after 24, 48 or 72 h), cells were incubated with MTT solution for 2 h. Then, the percentage of viability and inhibition was recorded by measuring the absorbance at 490 nm.

#### 2.6.3. Hoechst 33,342 Assay

Apoptosis was assessed by Hoechst 33,342 staining (Apoptosis-Hoechst staining kit; Beyotime Biotechnology, Jiangsu-China) on the basis of DNA fragmentation. Walker 256/B cells were seeded in triplicate with increasing concentrations of the ASE (0 (D0), 50 (D1) or 100 μg/mL (D2)) and incubated at 37 °C. Walker 256/B cells were immersed in 1.5 mL of methanol for 15 min then rinsed twice with phosphate-buffered saline (PBS). Walker 256/B cells were stained with Hoechst 33,342 (1 µg/mL) in a dark chamber for 10 min followed by two more rinses in phosphate-buffered saline (PBS). Walker 256/B cells were analyzed by fluorescence microscopy using 348 and 480 nm wavelengths for excitation and emission, respectively. For each well, 300 Walker 256/B cells were analyzed and apoptotic percentages were recorded after 24, 48 and 72 h of incubation.

### 2.7. Animal Farming and Surgical Procedure

Twenty-four male albino Wistar rats were divided into 4 groups (6 per group). The rats were housed at well-controlled standard conditions of temperature (22 ± 2 °C), humidity (50% ± 10%) and 12 h light cycle with standard food and water available ad libitum. Rats were allowed to acclimatize for 2 weeks before starting the experimental study. The experimental procedure involves the surgical operations.

Briefly, rats were anesthetized with intraperitoneal injection of ketamine (100 mg/kg of body weight (BW)) and xylazine (10 mg/kg BW). Superficial incisions were made in the skin after disinfection with ethanol (70%), followed by another incision along the patellar ligament to expose the femoral diaphysis with minimal damage. All rats received a slow intraosseous injection of 10 µL of Walker 256/B cells (5 × 10^4^ in 10 µL) or saline for metastasized or non-metastasized rats, respectively. To prevent any leakage of malignant cells outside of the femur, the drilled hole was sealed with orthopedic bone wax. The wound was, finally, closed by metal skin clips. Twenty days later, all rats were euthanized. Previous studies have shown that 20 days is a sufficient period to induce osteolytic bone metastases [9,23]. All procedures involving in vivo study and experimental protocols in rats were conducted in accordance with the Ethical Committee Guidelines for the care and use of laboratory animals of our institution. The person who conducted these procedures (R.B.) holds a FELASA (for Federation of European Laboratory Animal Science Associations) certificate. All experiments were conducted in accordance with guidelines and approval of the local ethical committee for care and use of laboratory animals (12/ES/15).

Before induction of bone metastases, the Walker 256/B cells’ viability was determined by trypan blue exclusion assay. In fact, at the appropriate time, the cells were trypsinized, neutralized, stained with 0.4% trypan blue solution and then counted using a hemocytometer. Viability was at least 99% before ascite development or bone metastases induction.

### 2.8. In Vivo Assays

#### 2.8.1. In Vivo Anticancer Activity of the AS Extract on Breast Cancer Skeletal Metastases

After surgical operations, rats were divided into four groups, as follows:

- Control (CTRL) group: composed of six control-operated rats that received an injection of physiological saline in the right femur.
- W256 group: composed of six metastasized rats that received an injection of malignant Walker 256/B cells (10^4^ in 10 µL) in the right femur.
- W256-ASE group: composed of six metastasized rats that received an injection of 5 × 10^4^ malignant Walker 256/B cells in the right femur as above and were treated with ASE. ASE, dissolved in olive oil (4 mg/mL), was administered daily by gavage at a final concentration of 200 mg/kg BW.
- ASE group: composed of six control-operated rats that received an injection of physiological saline in the right femur and were treated with ASE (200 mg/kg BW).

This group repartition was used to assess the effects of ASE on osteolytic bone metastases and tumoral angiogenesis due to malignant Walker 256/B cells’ intra-osseous inoculation. The same amount of olive oil, which was used to dissolve ASE, was given to other groups (CTRL and W256).

Standard histology and histomorphometry were used to assess osteolytic bone metastases using a 25-point integrating filter at ×200 magnification of sections stained with histo-enzymatic detection of tartrate-resistant acid phosphatase (TRAcP) and modified Goldner’s trichrome. Sections, of 7 µm thick, were cut dry, in parallel to the long axis of the femur, using a heavy-duty dry microtome (Polycut S Reichert Jung, Germany) equipped with 50° tungsten knife (Leica Polycut S, Rueil-Malmaison, France). The following bone histomorphometric parameters were assessed for each group: trabecular bone volume (BV/TV, %), osteoid volume and surface (OV/BV and OS/BS, respectively, %), osteoclasts number per bone area (N.Oc/B.Ar, mm^−2^) and eroded surface (ES/BS, %) [30]. These parameters were determined under light microscope using 50 µm separated sections. Histological slides were examined blindly.

#### 2.8.2. In Vivo Angiogenesis Study

Standard histological analysis and histomorphometry were used to assess tumoral angiogenesis using a 25-point integrating filter at ×400 magnification of the metaphysis. Previous studies, including those done in our laboratory, showed a reproducible bone loss-associated enhanced angiogenesis next to the growth plate [10,11]. The following angio-architectural histomorphometric parameters were assessed for each group: the distance of mean lumen diameter (MLD, in µm), wall thickness (WTh, µm) and the vessel number (VNb, µm), volume (VV, %) and separation (VSp, mm), and were determined under light microscope using sections with intervals of 50 µm. Histological slides were examined blindly.

### 2.9. Statistical Analyses

Data are expressed as the mean ± standard deviation (SD). The statistical analysis was assessed using GraphPad software package 8.3.1 (SPSS Inc., Chicago, IL, USA). The one-way analysis of variance (ANOVA) and the Tukey’s post hoc test were performed. The nominal statistical significance was considered with *p* < 0.05.

## 3. Results and Discussion

Skeletal metastasis is the main consequence of prostate and breast malignancies due to their high osteophily and proliferation index. They escape from the apoptotic cell death and their metastatic potential, angiogenesis plays a key role in cancer and metastatic development. In this study, ASE extract was subjected to phytochemical composition analysis, drug-likeness and pharmacokinetics assessments. Furthermore, the biological activity of ASE was assessed towards cancer cells’ proliferation, apoptotic potential, angiogenesis and osteolytic metastasis.

### 3.1. Phytochemical Composition: HR-LCMS and Bioinformatics’ Findings

**Figure 1:**
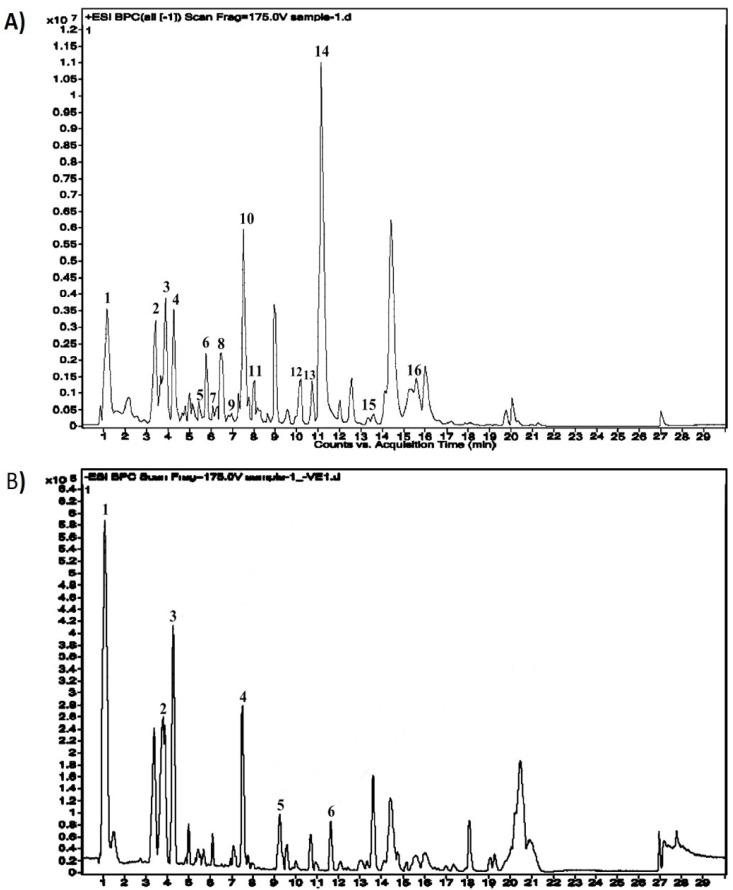
Phytochemical composition profile of Allium subhirsutum methanolic extract using the High Resolution-Liquid Chromatography Mass Spectroscopy (HR-LCMS) technique. (**A**) Positive peak (1). Pro Leu, (2). Cys Tyr Trp, (3). Glu Thr, (4). Asp Arg Tyr, (5). 2-methylene-5-(2,5dioxotetrahydrofuran-3-yl)-6-oxo--10,10-dimethylbicyclo [7:2:0] undecane, (6). (22S)-1alpha,22,25-trihydroxy-26,27-dimethyl-23,23,24,24-tetradehydro-24ahomovitaminD3/(22S)-1al, (7). L-4-Hydroxy-3-methoxy-amethylphenylalanine, (8). 1-nonadecanoyl-2-(5Z,8Z,11Z,14Z,17Zeicosapentaenoyl)-sn-glycerol, (9). TG(16:1(9Z)/17:2(9Z,12Z)/20: 5(5Z,8Z,11Z,14Z,17Z))[iso6], (10). 11alpha-acetoxykhivorin, (11) Methyl gamboginate, (12). C16 Sphinganine, (13). 4-Oxomytiloxanthin, (14). Sebacic acid, (15). Linolenoyl lysolecithin, (16). 3beta, 7alpha, 12alpha-Trihydroxy-5alpha-cholestan- 26-oic acid, (17). N-(2-hydroxyethyl) stearamide. (**B**) Negative peak (1). Asn Asn Asn, (2). His Asp, (3). Cepharanthine, (4). Asn Gln Ala, (5). 6 alpha-Hydroxy Castasterone, (6). 6-Deoxocastasterone. Abbreviations: Ala: Alanine, Arg: Arginine, Asn: Asparagine, Asp: Aspartic acid, Cys: Cysteine, Gln: Glutamine, Glu: Glutamic acid, His: Histidine, Leu: Leucine, Pro: Proline, Thr: Threonine, Tyr: Tyrosine.

Figure 1 exhibits a HR-LCMS chromatogram regarding *A. subhirsutum* methanolic extract. The different phytochemical compounds are shown in Table 1 and Table 2. As shown in Figure 1, the most abundant compound is 14, followed by 10, then 1, 3 and 4. Results are comparable to a previous study using LC-DAD-MS [31]. 

**Figure 2:**
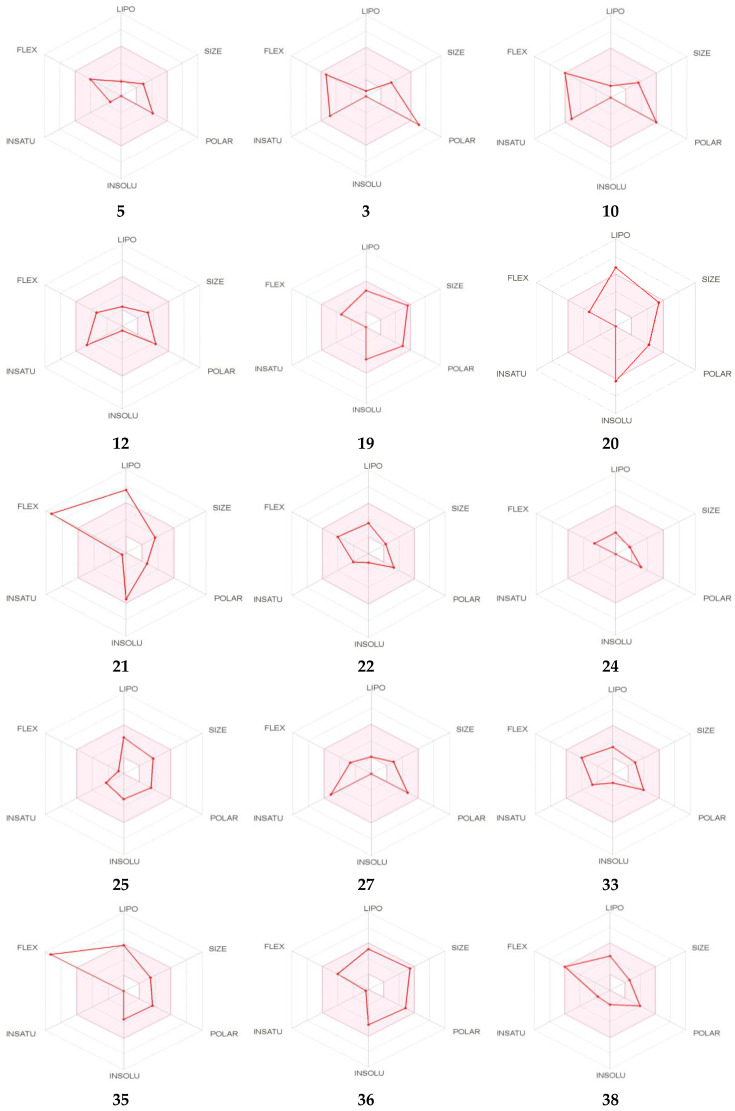

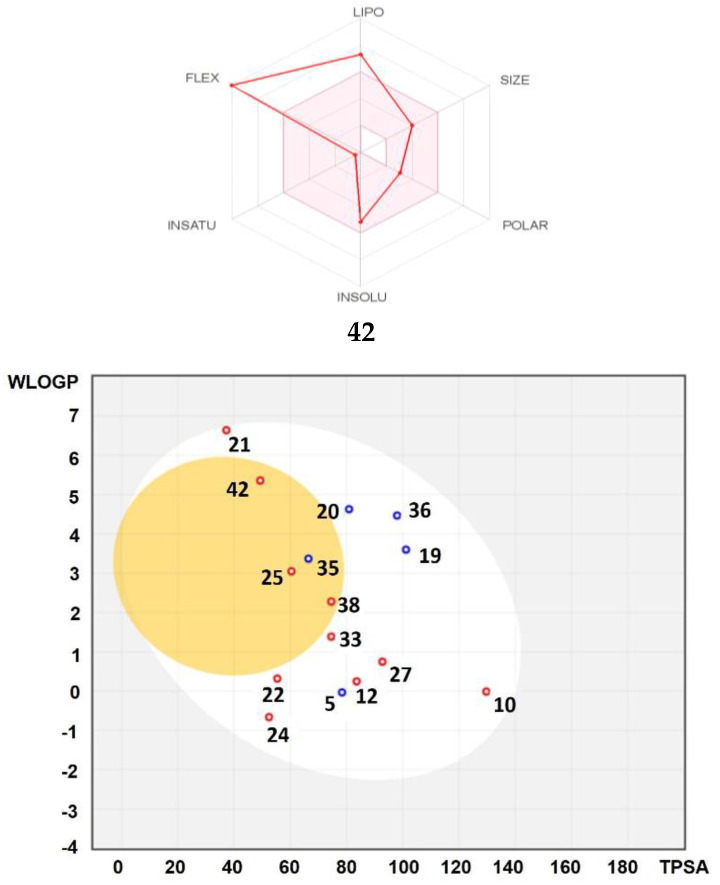
Bioavailability radar of identified compounds based on physicochemical indices ideal for oral bioavailability. LIPO, Lipophilicity: −0.7 < XLOGP3 < þ 5; SIZE, Molecular size: 150 g/mol < mol. wt. < 500 g/mol; POLAR, Polarity: 20 Å2 < TPSA < 130 Å2; INSOLU, Insolubility: 0 < Log S (ESOL) < 6; INSATU, Insaturation: 0.25 < Fraction Csp3 < 1; FLEX, Flexibility: 0 < Number of rotatable bonds < 9. The colored zone is the suitable physicochemical space for oral bioavailability (Top). Boiled-egg model of identified compounds (Bottom). ESOL: Estimating Aqueous Solubility from Molecular Structure, TPSA: topological polar surface area.

In order to provide the most promising compounds and reduce the risk of drug attrition in the late stage, the ADME parameters of the identified compounds were evaluated using SwissADME software. Herein, we have taken account only of analogs with the most potent ADME properties. As shown (Table 3), by obeying the Lipinski’s rule, the selected compounds manifest good oral absorption with good bioavailability score (0.55–0.56) and TPSA (topological polar surface area) values less than 140 Å^2^ (except 3), suggesting that they expected to be orally absorbed. Except those of 20 and 40, the rest of the compounds exhibited acceptable consensus Log Po/w. Regarding their pharmacokinetics properties, all selected compounds displayed high gastrointestinal (GI) properties (except 3) and were blood–brain barrier (BBB) permeant (except 23, 33, 36, 40). Some of them were predicted to be not P-gp substrate, giving them the property to have promising intestinal absorption with better bioavailability. The screened CYP (Cytochrome P450) enzyme isoforms (used in biotransformation of drugs and xenobiotics) data indicate that all selected compounds were found to be inhibitors of at least 3 isoenzymes among the tested CYP 1A2, CYP2C19, CYP2C9, CYP2D6 and CYP3A4 isoforms. Their negative skin permeability values allow them to be not permeable through the skin and therefore not suitable candidates for transdermal drug delivery. The drug-likeness behavior was assessed by the bioavailability radar (Figure 2) for oral bioavailability prediction. The results revealed that at least twelve compounds of the extract fall in the pink area of the polygon (Figure 2), suggesting good oral bioavailability.

The pharmacokinetic properties were investigated using the boiled-egg model, which allows for estimating of passive gastrointestinal absorption (GIA) and brain penetration (BBB) in function of the position of the molecules in the WLOGP versus TPSA referential (Figure 2). Results demonstrated that compounds possessing high probability to be passively absorbed by the gastrointestinal tract were 5, 10, 12, 18, 19, 20, 21, 22, 24, 25 and 31, with 5, 18, 19 and 31 being predicted to be substrates of the p-glycoprotein (PGP+), however the others were not substrates of the p-glycoprotein (PGP-). Analogs 23, 33, 36 and 40, having the capability of blood–brain barrier penetration, were located in the yolk, with only compound 33 predicted to be a substrate of the p-glycoprotein (PGP+).

### 3.2. Total Polyphenol and Flavonoid Contents and Antioxidant Properties

The total polyphenol and flavonoid contents of the *A. subhirsutum* methanolic extract are shown in Table 4. Results concerning the antioxidant content of ASE revealed promising phenolic and flavonoid levels. The colorimetric analysis regarding the total phenol and flavonoid contents exhibited 17.6 ± 0.8 mg GAE/g extract and 5.5 ± 0.8 mg QE/g extract, respectively. A recent study, published in 2020, showed similar results: 15.8 of TPC and 5.7 of TFC. Furthermore, the authors reported that the bulbs of AS possessed richer content in both TPC and TFC than the aerial parts of the same plant [16]. The study revealed that the TPC and TFC of ASE were much better than another garlic plant: *Allium nigrum* [16]. Nevertheless, in a comparative study on another wild edible garlic plant (*Allium nigrum* L.), the characterization of its phenolic profile revealed that the aerial part particularly, was richer [32].

In this study, the in vitro antioxidant activity of *A. subhirsutum* extract was investigated through two different assays: the reducing power and the antiradical activity against DPPH. The reducing power of ASE was increasing with the concentration of the extract (IC_50_ = 0.45 ± 0.02). The reducing power was reported to be concomitant with the antioxidant activity. In fact, reductones are able to terminate the reaction of free radical chains due to hydrogen atom donation [33,34]. Moreover, ASE demonstrated a potential antiradical scavenging activity (Table 4). This potential antioxidant effect was previously attributed to garlic species [35] and was able to protect cellular senescence in UVB-exposed skin cells [36] and potent biological activities related to the AS phytochemical composition [31].

### 3.3. In Vitro Findings: Cytotoxicity and Apoptotic Induction

**Figure 3:**
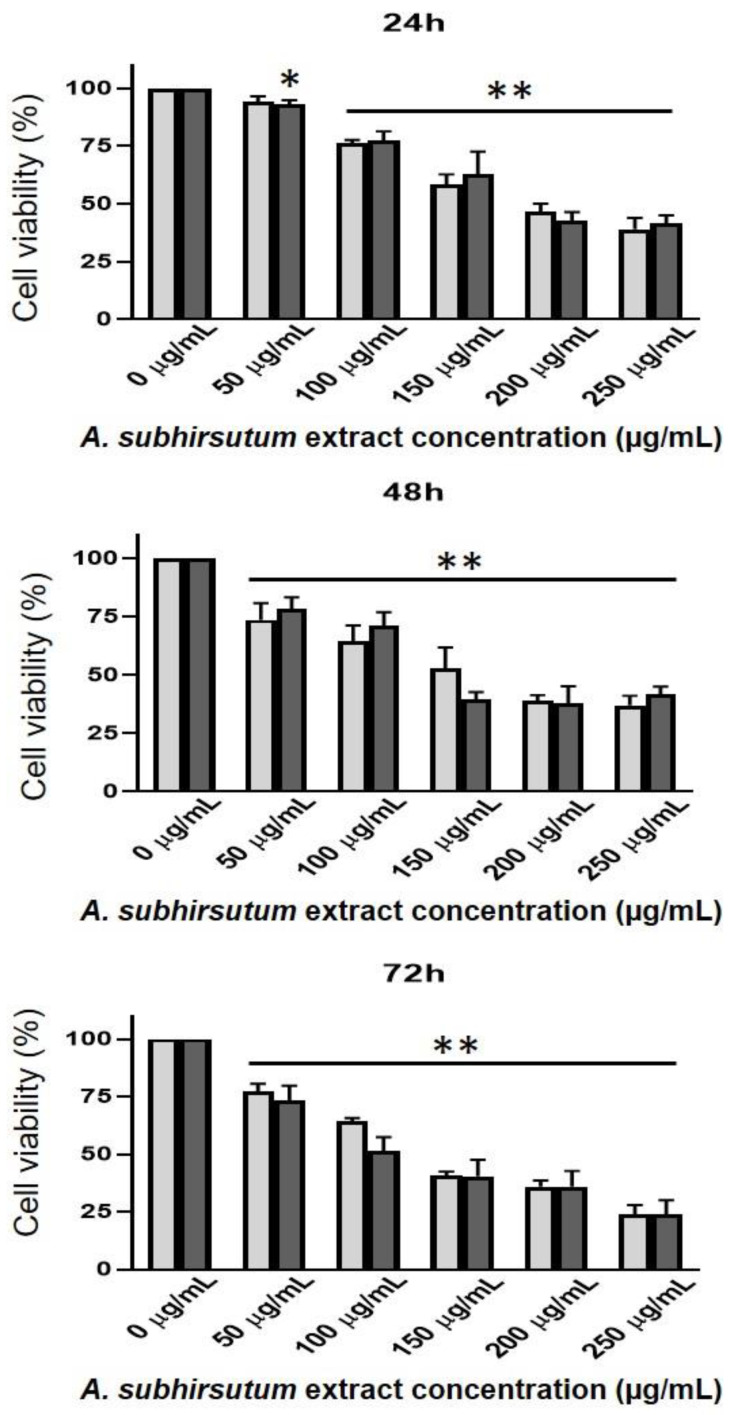
Time course and dose-response effects of *Allium subhirsutum* methanolic extract at different doses: 0, 50 and 100 µg/mL, on Walker 256/B and MatLyLu cells’ vitality. The cells were incubated for 24, 48 and 72 h. Cell viability was assessed using the MTT assay. Pure (100%) EthO was used as a positive control. Note that the effect is dose- and time-dependent. The most prominent effect was noticed at 72 h with the highest dose (250 µg/mL). Values represent mean ± standard deviation (SD) of *n* = 3. Statistical difference between the groups * *p* < 0.05, ** *p* < 0.01 following one-way analysis of variance (ANOVA) and Newman–Keuls post hoc tests. The experiment was run in triplicate.

Results of cells’ viability following treatment with ASE for 24, 48 and 72 h are given in Figure 3. Our results indicate that ASE possesses a significant protection against cancer cells’ proliferation. The effect was dose- and time-dependent. While 50 µg/mL exhibited no significant (*p* > 0.05) effect after 24 h on both Walker 256/B and MatLyLu cell lines, its effect was statistically significant (*p* < 0.01) after 48 and 72 h. These data clearly demonstrate that ASE acted as a chemo-preventive agent by reducing both malignant Walker 256/B and MatLyLu cell lines’ proliferation.

The IC_50_ for both cell lines was close to 150 µg/mL for the different screened concentrations. Several authors using different natural products in different cell lines outlined similar findings [34,37,38]. Their acceptable therapeutic effects together with their low cytotoxic potential led to deduce their efficient use against several diseases, including cancer [39]. Guesmi et al. [14] reported cell viability arrest in human multiple melanoma cell line (U266) following treatment with four different natural products.

**Figure 4:**
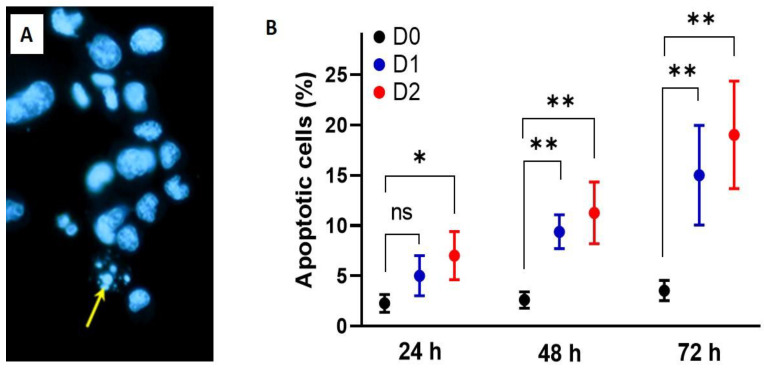
Time course and dose-response effects of *Allium subhirsutum* methanolic extract at different doses: 0, 50 and 100 µg/mL (D0, D1 and D2, respectively), on Walker 256/B cell apoptosis. The cells were incubated for 24, 48 and 72 h. Cell apoptosis was assessed using the Hoechst 33,342 assay (**A**). Note that D1 and D2 of AS extract were found to induce statistically high significant (* *p* < 0.001) increases after 48 h and the effect was more prominent at 72 h (**B**). Values represent mean ± SD of *n* = 3. Groups with different letters exhibit significant statistical difference at least at ** *p* < 0.05 following one-way ANOVA and Newman–Keuls post hoc tests. The experiment was run in triplicate.

The cytotoxic potential of ASE was apparently associated with reducing cells’ division and enhancing apoptosis. In the current study, apoptotic potential of ASE was assessed, on Walker 256/B cells, by Hoechst 33,342 assay on the basis of DNA fragmentation and therefore, apoptotic bodies. When compared with baseline (D0), a significant statistical increase (*p* < 0.05) of apoptotic bodies was noted in Walker 256/B treated only with D2 of ASE after 24 h. Both doses D1 and D2 of ASE were found to induce statistically high significant (*p* < 0.001) increases after 48 h and the effect was more prominent at 72 h (Figure 4). Hence, the apoptotic effect of *A. subhirsutum* was time- and concentration-dependent. Similarly, previous studies reported that exposure of cancer cells to plant natural products induced apoptotic cell death [40,41]. Millimouno et al. [40] reported that natural compounds could target apoptosis pathways in cancer. Das et al. [42] reported that garlic compounds activate cysteine proteases, which enhance apoptosis in human T98G and U87MG cells. Further methods of apoptosis detection, such as caspase activation or annexin double staining, may confirm these results.

In this study, the antiproliferative effect could be attributed to the chemical compounds revealed by HR-LCMS analysis. In fact, as shown in Table 1, *A. subhirsutum* extract exhibits a relevant and promising phytochemical composition following HR-LCMS assay. It includes, but is not limited to, flavonoid and isoflavonoid, isoprenoid, diterpene triepoxide (such as triptonide) and terpene (such as valtratum).

All these chemical compounds possess pharmacological activities. Basically, these natural compounds, such as the triptonide, were effective in inhibiting tumorigenicity and tumor growth in a wide variety of cancers, including pancreatic cancer and thyroid-induced metastases, by activating the tumor-suppressive Mitogen-Activated Protein Kinase Phosphatase (MAPKP) signaling pathway and via astrocyte-elevated gene-1, respectively [43,44]. Together, these phytochemical compounds might have better pharmacological properties than when separated. In fact, it has been reported that the whole-plant effects are usually much better than its active phytochemical compounds [13]. This might be the result of synergistic effects. Regarding the cytotoxic effect towards Walker 256/B and MatLyLu cells’ associated apoptotic potential, it can be deduced that *A. subhirsutum* could enhance anticancer drugs’ potency. In fact, to overcome the drug resistance in cancers, the use of natural products has recently gained great interest [37,45,46].

### 3.4. General In Vivo Findings

While no mortality was noticed during the experimental study, all the metastasized rats appeared seriously sick with hunched back, apathy and rough hair coat. Moreover, on the day of sacrifice, macro-anatomical examination revealed intraosseous cancer proliferation-associated bone osteolytic metastases in the metastasized rats (W256/B and W256/B-ASE groups). However, the effects were more prominent in the W256/B group. As follows, the different results of this study confirmed each other and proved the anticancer effects of the ASE.

These general observations noticed, before and after the day of euthanasia, have been previously reported in the same model of bone osteolytic metastases, confirming the reproducibility of the method.

### 3.5. In Vivo Findings: Bone Metastasis and Angiogenesis

Bone metastasis is one of the main complications of breast and prostate cancer. According to the X-ray phenotyping, breast- and prostate-induced bone metastases are categorized as osteolytic and osteosclerotic, respectively. In this study, methanolic extract of *A. subhirsutum* was used as a phytomedical substance to treat osteolytic metastases in a murine model due to Walker 256/B cells.

**Figure 5:**
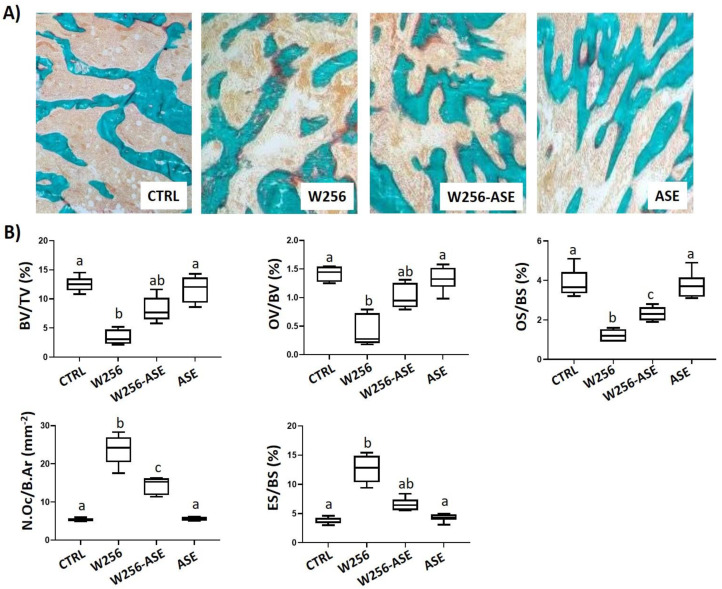
(**A**) Bone histological sections of the distal operated femurs from CTRL, W256, W256-ASE and ASE groups 20 days after surgery. The sections were stained with Goldner Modified Trichrome. W256/B slide exhibits cancellous and trabecular osteolysis once compared with CTRL and ASE. The inter-trabecular spaces are occupied with both hematopoietic cells together with tumor foci due to Walker 256/B cells’ inoculation. Note the alleviated trabecular network and bone quality in the W256-ASE histological section. Original magnification ×200. (**B**) Bone histomorphometric data for the distal operated femurs from CTRL, W256, W256-ASE and ASE. BV/TV: trabecular bone volume, OV/BV and OS/BS: osteoid volume and surface respectively, N.Oc/B.Ar: osteoclasts number per bone area, ES/BS: eroded surface. Values represent mean ± SD. Groups with different letters exhibit significant statistical difference at least *p* < 0.05 following one-way ANOVA and Newman–Keuls post hoc tests.

Histomorphometric analysis of the operated femur is presented in Figure 5. It indicates that the metastasized rats developed osteolytic metastases with haversian bone perforations and loss of trabecular bone in the operated femurs (reduced BV/TV, 12.53 vs. 3.38). While osteoid parameters (OV/BV, 1.41 vs. 0.4 and OS/BS, 3.86 vs. 1.21) were significantly decreased in W256 and W256-ASE groups, resorption parameters (N.Oc/BS, 5.36 vs. 23.68 and ES/BS, 3.9 vs. 12.65) were increased (Figure 5).

These finding suggest that malignant Walker 256/B cells interacted with the bone microenvironment and induced osteolytic metastasis by enhancing osteoclast activity and inhibiting osteoblast activity. These results parallel the common findings after malignant Walker256/B breast cancer cells’ injection in rats [47]. Thus, confirming the reproducibility of the method, which mimics the key features of human breast cancer skeletal metastases. However, these aspects were less prominent in W256-ASE and the extent lytic area was more limited once compared with the W256 group. They were restored towards normal ranges.

In fact, ASE increased the osteoblast activity, as assessed by OV/BV and OS/BS, and inhibited the osteoclast activity, as assessed by both ES/BS and N.Oc/B.Ar, and this could explain, even in part, the increased trabecular bone volume (BV/TV) in the W256-ASE group when compared with the W256 group. The protective effect of ASE is certainly related to the phytochemical profile of the extract and to its antioxidative effects. The effects of the natural products varied with the chemical composition [48]. The role of oxidative stress is associated with cancer development and therapy, including breast and prostate cancer [49]. Likewise, osteoclastogenesis is enhanced by reactive oxygen species. In this context, several osteolytic pathologies were associated with oxidizing stress and pain [50]. In this report, the rich content with bioactive compounds, as assessed by antioxidant activities, apoptosis and anti-proliferative effects, could interfere, in vivo, with the different compartments of bone metastatic environment. Thus, inhibiting the metastatic development.

**Figure 6:**
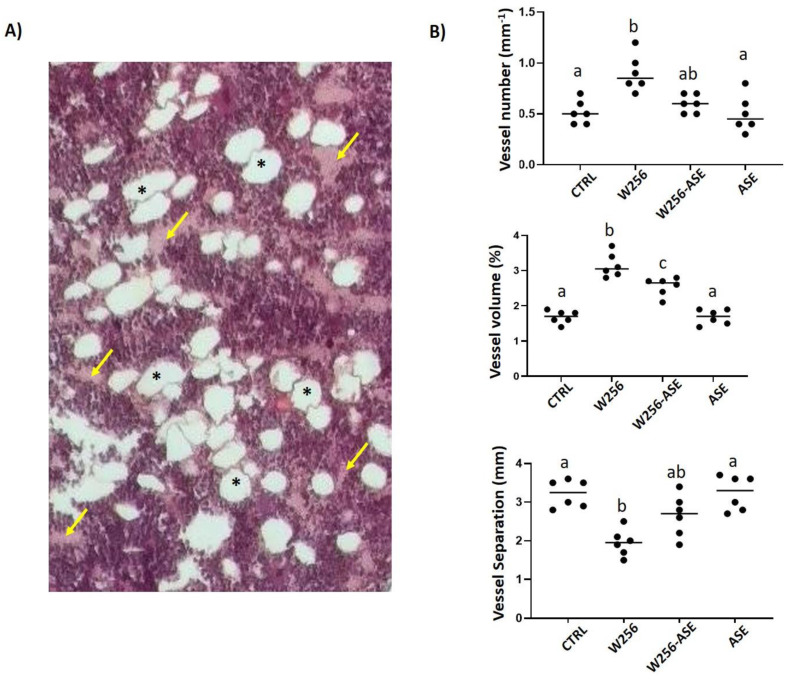
(**A**) Bone histological section of the distal operated femur from W256 rat 20 days after surgery. The cross-section was realized close to the tumoral foci, just below the growth plate. The section exhibits a high number of blood vessels (arrows) and adipocytes (asterisks) surrounded by a polymorph aspect of hematopoietic lineage and Walker 256/B cells. Original magnification ×200. (**B**) Angio-architecture of the distal operated femurs from CTRL, W256, W256-ASE and ASE 20 days after surgery. The angioarchitecture was assessed by two-dimensional (2D) histomorphometric analysis. Values represent mean ± SD. Groups with different letters exhibit significant statistical difference at least * *p* < 0.05 following one-way ANOVA and Newman–Keuls post hoc tests.

Data related to angiogenesis exhibit that there was a clear tendency for an enhanced tumor hypervascularity following in situ inoculation of Walker 256/B cells (reduction of V.Sp and increase of VN and V.V) (Figure 6). This enhanced angiogenesis is in parallel to the bone loss. In fact, the cancerous Walker 256/B cell line proliferation within the bone microenvironment is associated with increased Parathyroid Hormone-related Protein (PTHrP). PTHrP is one of the major stimulators of osteoclastogenesis and osteoclast resorbing activity. Taking into account that angiogenesis is crucial for cancer survival and development, intraosseous Walker 256/B proliferation is associated with new blood vessel structures supply. This is a hallmark of cancer invasion and metastases. The results of this study confirm previously reported data [11,23]. These new blood vessels may appear by pre-existing endothelial cells’ division or from angioblasts. Supplementary blood vessels invade the cancerous tissues for a localized support: oxygen and nutrient’s supply and metabolic waste removal [51,52,53]. This study provides the potential effect of AS for the treatment of cancer. Overall, ASE not only induces apoptosis and inhibits cancer proliferation and tumor osteolysis, but also, it considerably alleviates tumor angiogenesis. In fact, tumor osteolysis and angiogenesis were less evidenced in W256-ASE once compared with W256. The findings of the current study could explain the intensified use of the plant in recent years. The outlined effects are certainly due to the phytochemical composition of the plant. In this study, important levels of TPC and TFC were reported.

## 4. Conclusions

The current work outlines that ASE inhibits tumor angiogenesis, possesses cancer antiproliferative activity both in vitro and in vivo and induces apoptosis. The outlined effects could be related, even in part, to the richness content of the plant: particularly polyphenols and flavonoids, together with several other bioactive compounds, as assessed by HR-LCMS. The results confirm the *A. subhirsutum* ethno-pharmacological use for therapeutic claims, specifically chemo-preventive activity and inhibition of tumor cell growth. Moreover, AS can be exploited as a plant-based antioxidant and anticancer agent due to its biologically effective components. Its methanolic extract seems to be a promising candidate to further advance anticancer research. In extrapolation, *A. subhirsutum* intake might be helpful for alleviating bone malignancies and tumoral proliferation in humans.

## Figures and Tables

**Table 1:** Phytochemical composition of *A. subhirsutum* methanolic extract using the HR-LCMS technique.

N°	Compounds	Class of Compounds	RT	MW	Formula	[*m*/*z*]-	[*m*/*z*]+
1	Methyl N-(amethylbutyryl) glycine	Amino Acid	1.088	173.1041	C8 H15 N O3	-	156.1004
2	Bis (2-hydroxypropyl) amine	Amino alcohol	1.377	133.1113	C6 H15 N O2	-	156.0986
3	Cepharanthine	Alkaloid	4.265	606.2669	C37 H38 N2 O6	128.0394	-
4	2-methylene-5-(2,5dioxotetrahydrofuran-3-yl)-6-oxo--10,10-dimethylbicyclo [7:2:0] undecane	Amide	5.394	304.1626	C18 H24 O4	-	120.0795
5	(22S)-1alpha,22,25-trihydroxy-26,27-dimethyl-23,23,24,24-tetradehydro-24ahomovitamin D3/(22S)-1al	-	5.647	470.3446	C30 H46 O4	-	210.1465
6	L-4-Hydroxy-3-methoxy- amethylphenylalanine	Amino Acid	6.247	225.1012	C11 H15 N O4	-	206.0785
7	N-(2-fluro-ethyl) arachidonoyl amine	Fatty Amide	6.366	349.2812	C22 H36 F N O	-	332.2789
8	1-nonadecanoyl-2- (5Z,8Z,11Z,14Z,17Zeicosapentaenoyl)-sn-glycerol	Glycerolipid	6.544	656.5219	C42 H72 O5	-	210.1477
9	TG(16:1(9Z)/17:2(9Z,12Z)/20: 5(5Z,8Z,11Z,14Z,17Z))[iso6]	Glycerolipid	7.05	860.7058	C56 H92 O6	-	210.1471
10	L-4-Hydroxy-3-methoxy-amethylphenylalanine	Amino Acid	7.311	225.1012	C11 H15 N O4	-	206.0799
11	11 alpha-acetoxykhivorin	Limonoid	7.44	644.2711	C34 H44 O12	-	177.0531
12	Tuberonic acid	Octadecanoid	7.93	226.1192	C12 H18 O4	-	227.1282
13	Methyl gamboginate	-	8.226	662.2854	C39 H47 Cl O7	-	323.0934
14	Dihydrodeoxystreptomycin	Aminoglycoside antibiotic	8.968	567.2883	C21 H41 N7 O11	-	99.0444
15	6 alpha-Hydroxy Castasterone	Sterol Lipid	9.349	466.3618	C28 H50 O5	447.3427	-
16	C16 Sphinganine	Cationic Sphingoid	10.222	273.2653	C16 H35 N O2	-	274.2751
17	3beta,7alpha,12alpha-Trihydroxy-5alpha-cholestan-26-oic acid	Sterol Lipid	10.811	450.3325	C27 H46 O5	-	271.2014
18	4-Oxomytiloxanthin	Isoprenoid	10.814	612.3855	C40 H52 O5	-	253.1909
19	Sebacic acid	Fatty Acyl	11.057	202.1211	C10 H18 O4	-	227.1261
20	Tuberonic acid	Octadecanoid	11.101	226.1203	C12 H18 O4	-	227.1230
21	6-Deoxo castasterone	Sterol Lipid	11.65	450.366	C28 H50 O4	431.3475	-
22	Linolenoyl lysolecithin	-	13.48	517.3154	C26 H48 N O7 P	-	184.0723
23	19-Amino-16-hydroxy-16-oxido-10-oxo-11,15,17-trioxa-165-phosphanonadecan-13-ylundecanoate GPETn(10:0/11:0)[U]	Fatty Acyls	14.714	537.3419	C26 H52 N O8 P	-	184.0718
24	3beta,7alpha,12alpha-Trihydroxy-5alpha-cholestan- 26-oic acid	Sterol Lipid	15.276	450.3329	C27 H46 O5	-	271.2038
25	N-(2-hydroxyethyl)stearamide	Lipid	19.734	327.3122	C20 H41 N O2	-	57.0693
26	2,2-difluoro-hexadecanoic acid	Fatty Acid	26.968	292.2227	C16 H30 F2 O2	309.1819	-

RT: retention time, MW: molecular weight, [*m*/*z*]- and [*m*/*z*]+: negative and positive mass divided by charge numbers.

**Table 2:** Peptide-like proteins identified by the HR-LCMS technique in *A. subhirsutum* methanolic extract.

N°	Compounds	Class of Compounds	RT	MW	Formula	[*m*/*z*]-	[*m*/*z*]+
27	Tyr Trp Phe	Small Peptide	0.979	514.2104	C29 H30 N4 O5	50.0155	-
28	Asn Asn Asn	Small Peptide	1.053	360.1374	C12 H20 N6 O7	89.0278	-
29	Pro Leu	Small Peptide	1.379	228.1455	C11 H20 N2 O3	-	229.1516
30	Cys Tyr Trp	Small Peptide	3.335	470.1557	C23 H26 N4 O5 S	-	145.0292
31	His Asp	Small Peptide	3.648	270.0931	C10 H14 N4 O5	56.0320	-
32	Glu Thr	Small Peptide	3.729	248.1015	C9 H16 N2 O6	-	229.0795
33	Thr Asp Asn	Small Peptide	4.031	348.134	C12 H20 N4 O8	-	170.0204
34	Cys Tyr Trp	Small Peptide	4.054	470.1559	C23 H26 N4 O5 S	-	120.0791
35	Phe Glu	Small Peptide	4.211	294.1201	C14 H18 N2 O5	-	120.0805
36	Asp Arg Tyr	Small Peptide	4.264	452.2018	C19 H28 N6 O7	-	278.0983
37	Phe Pro	Small Peptide	4.518	262.1305	C14 H18 N2 O3	-	146.0578
38	Val Ser Cys	Small Peptide	6.772	307.1227	C11 H21 N3 O5 S	-	128.0688
39	Asn Gln Ala	Small Peptide	7.526	331.1507	C12 H21 N5 O6	148.0574	-
40	Val Glu Asp	Small Peptide	7.59	361.151	C14 H23 N3 O8	-	177.0529
41	Gly Tyr Lys	Small Peptide	7.995	366.19	C17 H26 N4 O5	-	227.1255
42	Lys Arg Lys	Small Peptide	15.311	428.3053	C18 H38 N8 O4	-	271.2035

**Table 3:** Absorption, distribution, metabolism and excretion (ADME) properties of identified compounds.

Entry	5	3	10	12	19	20	21	22	24	25	27	33	35	36	38	42
**Physicochemical Properties/Lipophilicity/Drug-likeness**																
**Molecular Weight**	228.29	270.24	294.3	262.3	466.69	450.69	292.41	173.21	133.19	304.38	225.24	226.27	273.45	450.65	202.25	327.55
**Num. heavy atoms**	16	19	21	19	33	32	20	12	9	22	16	16	19	32	14	23
**Num. arom. heavy atoms**	0	5	6	6	0	0	0	0	0	0	6	0	0	0	0	0
**Fraction Csp3**	0.82	0.4	0.36	0.43	1	1	0.94	0.75	1	0.72	0.36	0.67	1	0.96	0.8	0.95
**Num. rotatable bonds**	6	8	9	5	5	5	14	6	4	1	4	6	14	6	9	19
**Num. H-bond acceptors**	4	7	6	4	5	4	4	3	3	4	5	4	3	5	4	2
**Num. H-bond donors**	3	5	4	2	5	4	1	1	3	0	3	2	3	4	2	2
**Molar Refractivity**	64.37	61.65	74.31	74.43	133.54	132.38	80.94	44.86	36.08	83.25	58.86	60.34	84.06	128.18	53.73	102.42
**TPSA** (Å^2^)	78.43	158.4	129.72	83.63	101.15	80.92	37.3	55.4	52.49	60.44	92.78	74.6	66.48	97.99	74.6	49.33
**Consensus Log *P*_o/w_**	0.04	3.41	−0.23	0.32	3.27	4.66	5.59	0.88	−0.07	2.9	−0.12	1.23	3.64	3.74	1.88	5.5
**Lipinski’s Rule**	Yes	Yes	Yes	Yes	Yes	Yes	Yes	Yes	Yes	Yes	Yes	Yes	Yes	Yes	Yes	Yes
**Bioavailability Score**	0.55	0.55	0.56	0.55	0.55	0.55	0.56	0.55	0.55	0.55	0.55	0.56	0.55	0.56	0.56	0.55
**Pharmacokinetics**																
**GI absorption**	High	High	High	High	High	High	High	High	High	High	High	High	High	High	High	High
**BBB permeant**	No	Yes	No	No	No	No	No	No	No	Yes	No	No	Yes	No	Yes	Yes
**P-gp substrate**	Yes	No	No	No	Yes	Yes	No	No	No	No	No	No	Yes	Yes	No	No
**CYP1A2 inhibitor**	No	Yes	No	No	No	No	Yes	No	No	No	No	No	No	No	No	Yes
**CYP2C19 inhibitor**	No	No	No	No	No	No	No	No	No	No	No	No	No	No	No	No
**CYP2C9 inhibitor**	No	No	No	No	No	No	Yes	No	No	No	No	No	No	No	No	No
**CYP2D6 inhibitor**	No	No	No	No	No	No	No	No	No	No	No	No	Yes	No	No	No
**CYP3A4 inhibitor**	No	No	No	No	No	No	No	No	No	No	No	No	No	No	No	No
**Log Kp (cm/s)**	−9.4	−4.8	−10.23	−8.87	−7.12	−4.56	−2.64	−6.75	−7.69	−6.51	−9.05	−7.41	−4.62	−6.44	−6.04	−3.14

GI: gastro intestinal, BBB: blood-brain barrier, LIPO, Log *Po*/*w*: partition coefficient between n-octanol and water, P-gp: permeability of glycoprotein, CYP: Cytochrome P450.

**Table 4:** Antioxidant content and activities of *Allium subhirsutum* methanolic extract.

*Allium subhirsutum* Methanolic Extract			
Phytochemical Composition		Antioxidant Activities	
TPC (mg GAE/g Extract)	TFC (mg QE/g Extract)	Reducing Power IC_50_ (mg/mL)	DPPH IC_50_ (mg/mL)
17.6 ± 0.8	5.5 ± 0.8	0.45 ± 0.02	24.3 ± 1.9

TPC: The total phenolic content, TFC: total flavonoid content, GAE: gallic acid equivalent, QE: quercetin equivalent, IC_50_: Half-maximal inhibitory concentration.
